# Necessary Condition Analysis: Type I Error, Power, and Over-Interpretation of Test Results. A Reply to a Comment on NCA. Commentary: Predicting the Significance of Necessity

**DOI:** 10.3389/fpsyg.2019.01493

**Published:** 2019-07-24

**Authors:** Jan Dul, Erwin van der Laan, Roelof Kuik, Maciej Karwowski

**Affiliations:** ^1^Rotterdam School of Management, Erasmus University, Rotterdam, Netherlands; ^2^Department of Historical and Educational Sciences, Institute of Psychology, University of Wrocław, Wrocław, Poland

**Keywords:** Necessary Condition Analysis, NCA, null hypothesis testing, alternative hypothesis, significance, power, type I error, *p*-value

We reply to Sorjonen and Melin (2019) article “Predicting the significance of necessity” that is a comment on a recently proposed statistical test for Necessary Condition Analysis (Dul et al., in press). Necessary Condition Analysis (NCA) is a method that draws a ceiling line on top of the data in an XY scatter plot (Dul, 2016). This line represents the level of X that is necessary but not sufficient for a given level of Y^1^. The empty space above the line is the necessity effect size. The statistical test for NCA is a null hypothesis test that detects the randomness of the empty space. It is a permutation test^2^ that produces an estimate of the *p*-value and “…is intended to answer the question: ‘Can the observed effect size be the result of random chance?' by responding: ‘Yes, but with probability smaller than *p*.”' (Dul et al., in press, p. 2). Dul et al. (in press) show by simulations and by referring to a mathematical proof that the test is valid for identifying randomness, hence for helping researchers to avoid type I error (rejecting the null hypothesis when the null is true).

Sorjonen and Melin (2019) comment on this test aims to give “indications of the power of the method as well as risk for type 1-errors.” (Sorjonen and Melin, 2019, p. 2). They use simulations with different true alternative hypotheses: H1 when there is a necessity effect (upper left corner is empty), H2 when there is a necessity effect and a sufficiency effect (the upper left corner is empty and the lower right corner is empty), and H3 when there is a sufficiency effect (lower right corner is empty). Inspection of the simulation results indeed shows (again) that when all effect sizes are zero (null is true) the test for NCA correctly identifies randomness. Sorjonen and Melin (2019) seem to acknowledge this quality of the test for NCA: “Without any true population sufficiency effect, NCA did not seem to result in more type 1-errors than expected, i.e., 5%” (p. 5), at least for the case that the necessity effect is also absent (ensuring that the null is true). The simulation results also show (for the first time) that the test has high power (rejecting the null when the alternative is true): When an alternative is true the test correctly identifies non-randomness. Hence, the simulations show that the statistical test for NCA is not only valid regarding type I error but has also high power.

However, when Sorjonen and Melin (2019) discuss on the simulation results they deviate from statistical definitions of power and type I error and make an over-interpretation of test results of a null hypothesis test. In their discussion of the power, they use necessity as the only alternative hypothesis (H1). But the test also rejects, and should reject, the null when the other alternatives are true (H2 and H3). Sorjonen and Melin (2019) do not mention this as also an indication of the power of the test. Instead they call the latter result a “type I error.” For example, they state (p. 3) that “while sample size had no effect on the probability to get a significant observed necessity effect, i.e., the risk for type 1-error, this risk increased with increased true population sufficiency effect.” This interpretation of “type I error” does not correspond to the definition in statistics, which is only defined when the *null* is true, not when an alternative is true. It is a common misunderstanding to interpret a rejection of the null hypothesis as the acceptance of a *specific* alternative hypothesis, in this case necessity. This misinterpretation is formulated by Szucs and Ioannidis (2017, p. 8) as follows: “A widespread misconception … is that rejecting H0 allows for accepting a *specific* H1.…This is what most practicing researchers do in practice when they reject H0 and argue for their specific H1 in turn” [emphasis in the original]. Also Sorjonen and Melin (2019) comment on the statistical test for NCA focuses on this incorrect over-interpretation of test results. When referring to the high power of NCA's test, they state: “Of course, this apparent high power of NCA could be seen as a positive characteristic. However, one might also become a bit worried by the ease with which people wanting to claim that X is a necessary condition for Y can overcome the obstacle of significance.” In this worry, they assume that people make the same incorrect over-interpretation of having a significant (small *p*-value) *necessity* result, whereas they truly have found a significant *non-random* result.

The statistical test for NCA is a valid and powerful “minimum statistical test” (Dul et al., in press, p. 8) that can test the randomness of an empty space in the upper left corner of a XY scatter plot: not more, not less. It may seem disappointing that a null hypothesis test like the one for NCA can only test whether a result is due to randomness or not, and cannot test for a specific alternative hypothesis. However, this is inherent to null hypothesis testing. For direct testing of a necessity hypothesis, other statistical approaches need to be developed, such as Bayesian approaches. Such approaches are currently not available for NCA and may be a topic for future research.

## Author Contributions

JD wrote the first draft and revisions of the manuscript. RK, EvdL, and MK contributed to successive revisions.

### Conflict of Interest Statement

The authors declare that the research was conducted in the absence of any commercial or financial relationships that could be construed as a potential conflict of interest.

## Footnotes

^1^It is not “paradoxical,” as suggested by Sorjonen and Melin (2019), that a ceiling line and a floor line are both present at the same time. The ceiling line represents that a certain X value is necessary but not sufficient for a certain Y value on the ceiling line. The floor line represents that a certain X value is sufficient but not necessary for a certain Y value on the floor line.

^2^The permutation test and NCA's significance test are also called “randomness tests.” The null sampling distribution is obtained by shuffling Y values over X values, or by shuffling X values Y values, which, contrary to what Sorjonen and Melin (2019) claim, gives identical results.
